# CCTA-based CABG SYNTAX Score: a tool to evaluate completeness of coronary segment revascularization after bypass surgery

**DOI:** 10.1007/s10554-023-02978-9

**Published:** 2023-11-03

**Authors:** Nozomi Kotoku, Patrick W. Serruys, Shigetaka Kageyama, Scot Garg, Shinichiro Masuda, Kai Ninomiya, Juan B. Grau, Himanshu Gupta, Vikram Agarwal, Marie-Angèle Morel, Torsten Doenst, Ulrich Schneider, Kaoru Tanaka, Mark LaMeir, Saima Mushtaq, Pontone Gianluca, Giulio Pompilio, Ulf Teichgräber, John Puskas, Jagat Narula, Johan de Mey, Daniele Andreini, Yoshinobu Onuma

**Affiliations:** 1 Department of Cardiology, University of Galway, Galway, Ireland; 2 Department of Cardiology, Royal Blackburn Hospital, Blackburn, UK; 3 Department of Cardiothoracic Surgery, The Valley Hospital, Ridgewood, NJ USA; 4 Cardiac Imaging, Valley Health System, Ridgewood, NJ USA; 5 Department of Cardiology, Icahn School of Medicine at Mount Sinai, Mount Sinai Morningside, New York, NY USA; 6 Department of Cardiothoracic Surgery, Friedrich-Schiller-University Jena, University Hospital, Jena, Germany; 7 Department of Radiology, Universitair Ziekenhuis Brussel, Brussels, Belgium; 8 Cardiac Surgery Department, Universitair Ziekenhuis Brussel, VUS, Brussels, Belgium; 9 Departments of Perioperative Cardiology and Cardiovascular Imaging, Centro Cardiologico Monzino, IRCCS, Milan, Italy; 10 Department of Biomedical Surgical and Dental Sciences, University of Milan, Milan, Italy; 11 Department of Radiology, Friedrich Schiller University, Jena University Hospital, Jena, Germany; 12 Department of Cardiovascular Surgery, Mount Sinai Morningside Hospital, New York, USA; 13 University of Texas Health Science Center at Houston, Houston, TX USA; 14 Division of Cardiology and Cardiac Imaging, IRCCS Galeazzi Sant’Ambrogio, Milan, Italy; 15 Department of Biomedical and Clinical Sciences, University of Milan, Milan, Italy; 16 Cardiovascular Research Centre for Advanced Imaging and Core Lab (CORRIB) Research Centre, University of Galway, University Road, Galway, H91 TK33 Ireland

**Keywords:** Completeness of revascularization, Coronary artery bypass graft, Coronary artery disease, Coronary computed tomographic angiography, SYNTAX Score

## Abstract

To describe the updated coronary computed tomographic angiography (CCTA)-based coronary artery bypass graft (CABG) anatomic SYNTAX Score (aSS) and assess its utility and reproducibility for assessing the completeness of revascularization after CABG. The CCTA-CABG aSS is a visual assessment using CCTA post-CABG which quantifies the failure in effectively grafting stenotic coronary segments, and therefore assesses the completeness of surgical revascularization. It is calculated by subtracting the aSS of successfully anastomosed coronary segments from the aSS of the native coronary tree. The inter-observer reproducibility of the CCTA-CABG aSS was evaluated in 45 consecutive patients with three-vessel disease with or without left main disease who underwent a CCTA 30 days (± 7 days) after CABG. The CCTA-CABG aSS was evaluated in 45 consecutive patients with 117 bypass grafts and 152 anastomoses. The median native coronary aSS was 35.0 [interquartile range (IQR) 27.0–41.0], whilst the median CCTA-CABG aSS was 13.0 (IQR 9.0–20.5). The inter-observer level of agreement for the native coronary aSS and the CCTA-CABG aSS were both substantial with respective Kappas of 0.67 and 0.61. The CCTA-CABG aSS was feasible in all patients who underwent CABG for complex coronary artery disease with substantial inter-observer reproducibility, and therefore can be used to quantify the completeness of revascularization after CABG.

**Supplementary Information:**

The online version contains supplementary material available at 10.1007/s10554-023-02978-9.

## Introduction

The angiographic coronary artery bypass graft (CABG) anatomic SYNTAX Score (aSS) was developed and used to evaluate the patency of bypass grafts at mid-term follow-up after CABG in the LE MANS cohort of the SYNTAX trial [1]. Since it assessed residual and non-revascularized lesions after CABG, it was able to quantify the completeness of surgical revascularization.

Computation of the CABG aSS consists of two steps: the first being to calculate the aSS of the native coronary arteries, and the second to evaluate the patency of the bypass grafts; wherein if a stenotic lesion in a coronary segment is successfully bypassed, then the segment’s weighting points are subtracted from the aSS of the native circulation. In cases of narrowed or occluded bypass grafts, the deduction of points is reduced or nullified.

The diagnostic accuracy of coronary computed tomographic angiography (CCTA) has improved through technological advancements [2], and it is now commonly used as a first-line investigation of acute and chronic chest pain to rule out coronary artery disease (CAD), whilst in patients with complex CAD it is being increasingly used for diagnosis and decision-making [3, 4]. In addition, instead of invasive coronary angiography (ICA), CCTA can be used to calculate the aSS of native coronary arteries [5].

CCTA is an established imaging modality to evaluate bypass grafts [6], with high diagnostic performance in detecting bypass graft stenoses due to their larger diameter, minimal calcification, and lower susceptibility to cardiac motion artefact compared to native coronary arteries [7]. Occasionally images can be hampered by the metallic clips used for clamping side branches as these can produce artefactual “shadowing” of luminal structures [6]. A meta-analysis, investigating 2482 grafts in 959 patients, reported a respective sensitivity and specificity for the detection of obstructive graft lesions (> 50% diameter stenosis on ICA) using CCTA of 0.98 [95% confidence intervals (CI) 0.97–0.99] and 0.98 (0.96–0.98) with an area under the curve of 0.99 [8]. Mushtaq et al. demonstrated that in patients post CABG, a whole-heart coverage CT scanner allows evaluation of grafts and native coronary arteries with high interpretability and low radiation exposure, even in patients with atrial fibrillation and/or high heart rate [6].

Given the high diagnostic accuracy of CCTA for detecting graft and native coronary artery stenoses, it is a suitable modality to compute the CABG aSS. The purpose of this methodological report is, therefore, to describe the CCTA-CABG aSS, together with assessing its utility and reproducibility for evaluating the completeness of revascularization after CABG.

## Methods

### CCTA-based CABG-aSS

The CCTA-CABG aSS was evaluated using post-CABG CCTA, which visualised the entire native coronary tree and the full length of all bypass grafts [e.g., left internal mammary artery (LIMA)]. Typically, an additional acquisition was needed to image the origin of the IMA, whilst a three-dimensional volume rendering image was useful to understand the anatomy of the bypass grafts and their topological relationship with the native coronary arteries, whereas curved multiplanar reconstructions enabled luminal assessment of any stenoses. The score was calculated following the sequential steps:

1. The CCTA-aSS of the native coronary arteries was calculated as a summation of lesion-based aSS (Table 1) [5]. The SS algorithm was used to score all coronary lesions ≥ 50% in diameter stenosis by visual estimation, in vessel segments  ≥ 1.5 mm. The aSS was calculated multiplication factor related to lesion location (i.e., segment-weighing point) and severity (× 2 for obstructive lesion, × 5 for occluded lesion), and adverse lesion characteristics led to the addition of points, which included feature of total occlusions, presence of bifurcation or trifurcation disease, side branch angulation, aorto-ostial lesion, severe tortuosity, lesion length > 20 mm, heavy calcification, thrombus and diffuse or small vessel disease.

**Table 1:** Segment-weighting incorporated into the aSS for calculation of native coronary aSS

Segment		Right dominance	Left dominance
1	RCA proximal	1	0
2	RCA mid	1	0
3	RCA distal	1	0
4	Posterior descending artery	1	n/a
16	Posterolateral branch from RCA	0.5	n/a
16a		0.5	n/a
16b		0.5	n/a
16c		0.5	n/a
5	Left main	5	6
6	LAD proximal	3.5	3.5
7	LAD mid	2.5	2.5
8	LAD apical	1	1
9	First diagonal	1	1
9a		1	1
10	Second diagonal	0.5	0.5
10a		0.5	0.5
11	Proximal circumflex artery	1.5	2.5
12	Intermediate/anterolateral artery	1	1
12a	Obtuse marginal	1	1
12b		1	1
13	Distal circumflex artery	0.5	1.5
14	Left posterolateral	0.5	1
14a		0.5	1
14b		0.5	1
15	Posterior descending artery	n/a	1
Segment-weighting multiplication factor for calculation of native coronary aSS			
Obstructive lesion: 50–99%		×2 segment-weighting	
Occluded lesion: 100%		×5 segment-weighting	

*aSS* anatomical SYNTAX Score, *LAD* left artery descending artery, *n/a* not applicable, *RCA* right coronary artery

**Fig. 1:**
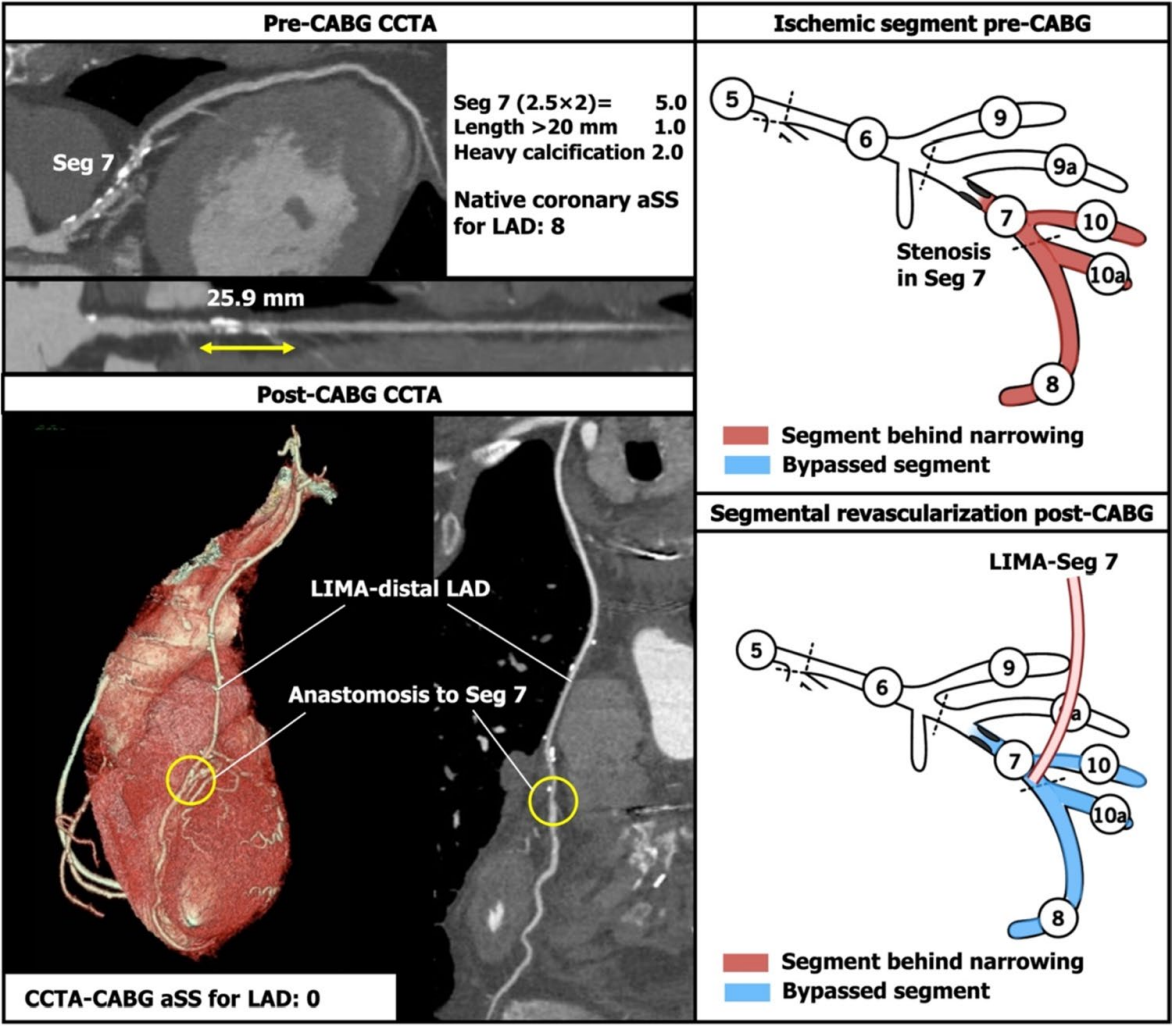
Example of points subtracted from the native coronary aSS in a left anterior descending artery (LAD) with a single lesion. If there was a lesion in segment (Seg) 7 and a bypass was placed on the distal LAD, the aSS of Seg 7 would be subtracted. *aSS* anatomical SYNTAX Score, *CABG* coronary artery bypass graft, *CCTA* coronary computed tomographic angiography, *LAD* left artery descending artery, *LIMA* left internal mammary artery

**Fig. 2:**
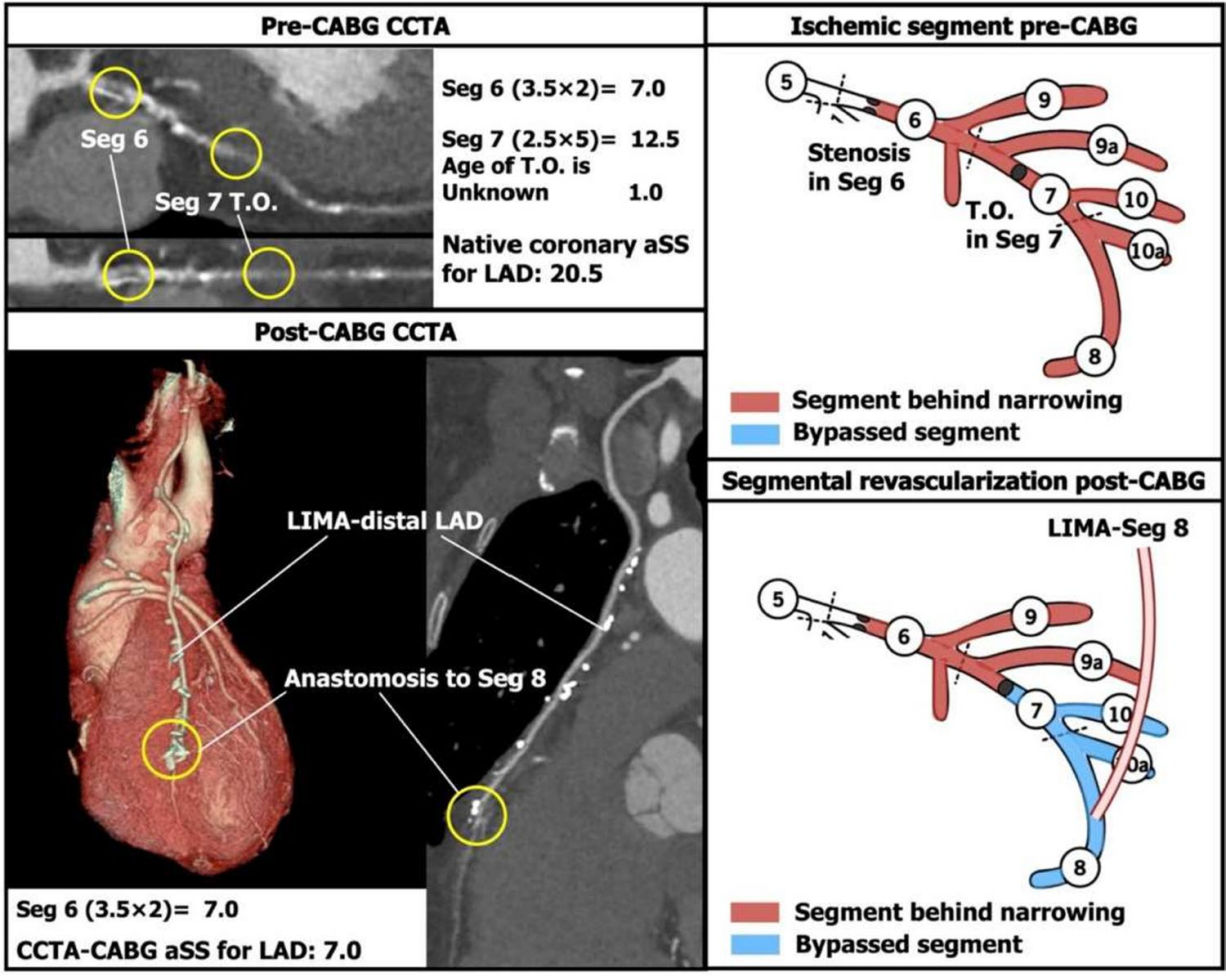
Example of the points subtracted from the native coronary aSS in a left anterior descending artery (LAD) with sequential lesions. If there were sequential lesions in segment (Seg) 6 and 7, and a bypass graft was anastomosed in the distal LAD, only the aSS of Seg 7 would be subtracted. *T.O.* total occlusion. Other abbreviations as in Fig. 1

**Fig. 3:**
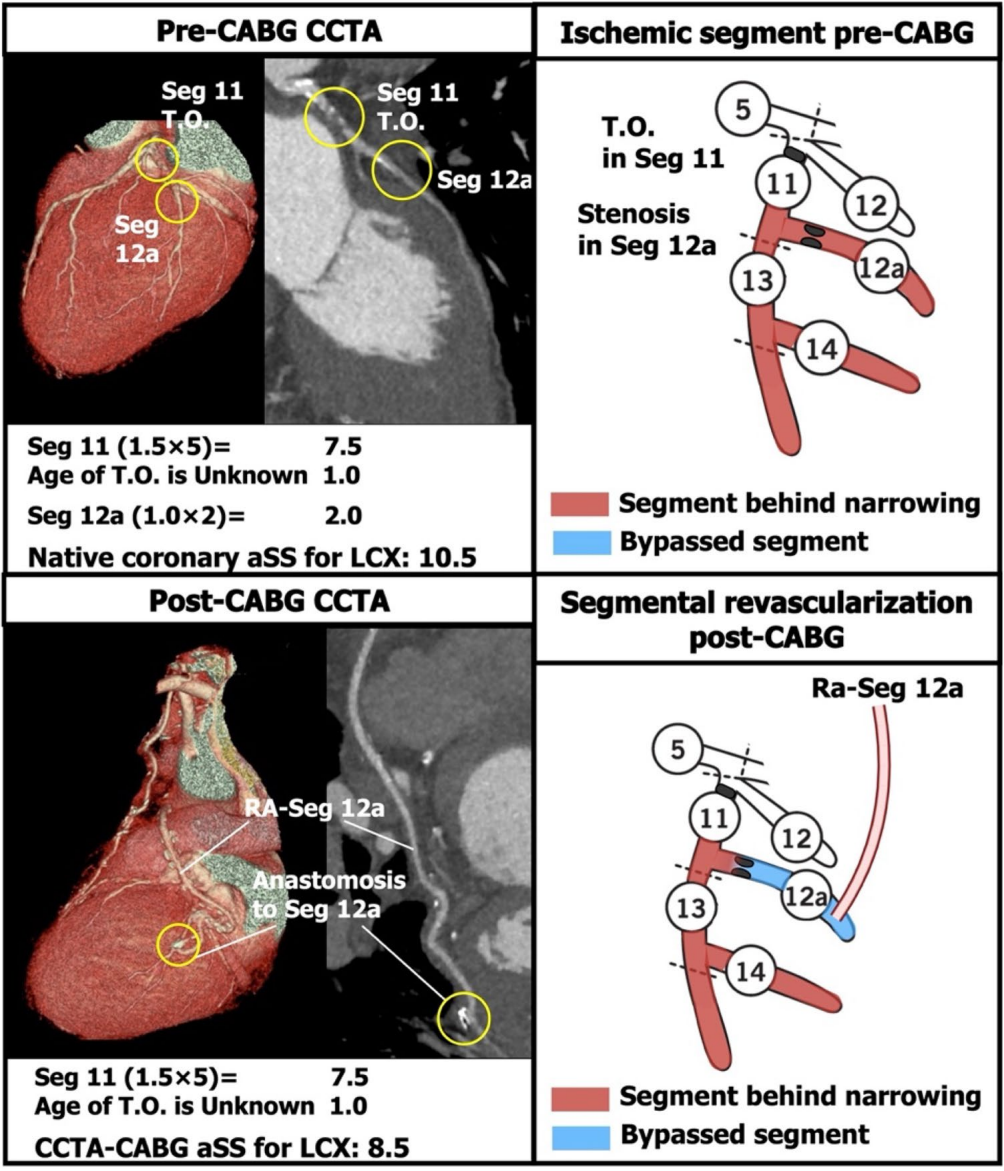
Example of subtraction of points from the native coronary aSS in a left circumflex (LCX) with sequential lesions. If sequential lesions in segment (Seg) 11 and Seg 12a were bypassed with a single anastomosis to Seg 12a, the aSS points of Seg 11 remain. *Ra* radial artery, *T.O.* total occlusion. Other abbreviations as in Fig. 1

**Fig. 4:**
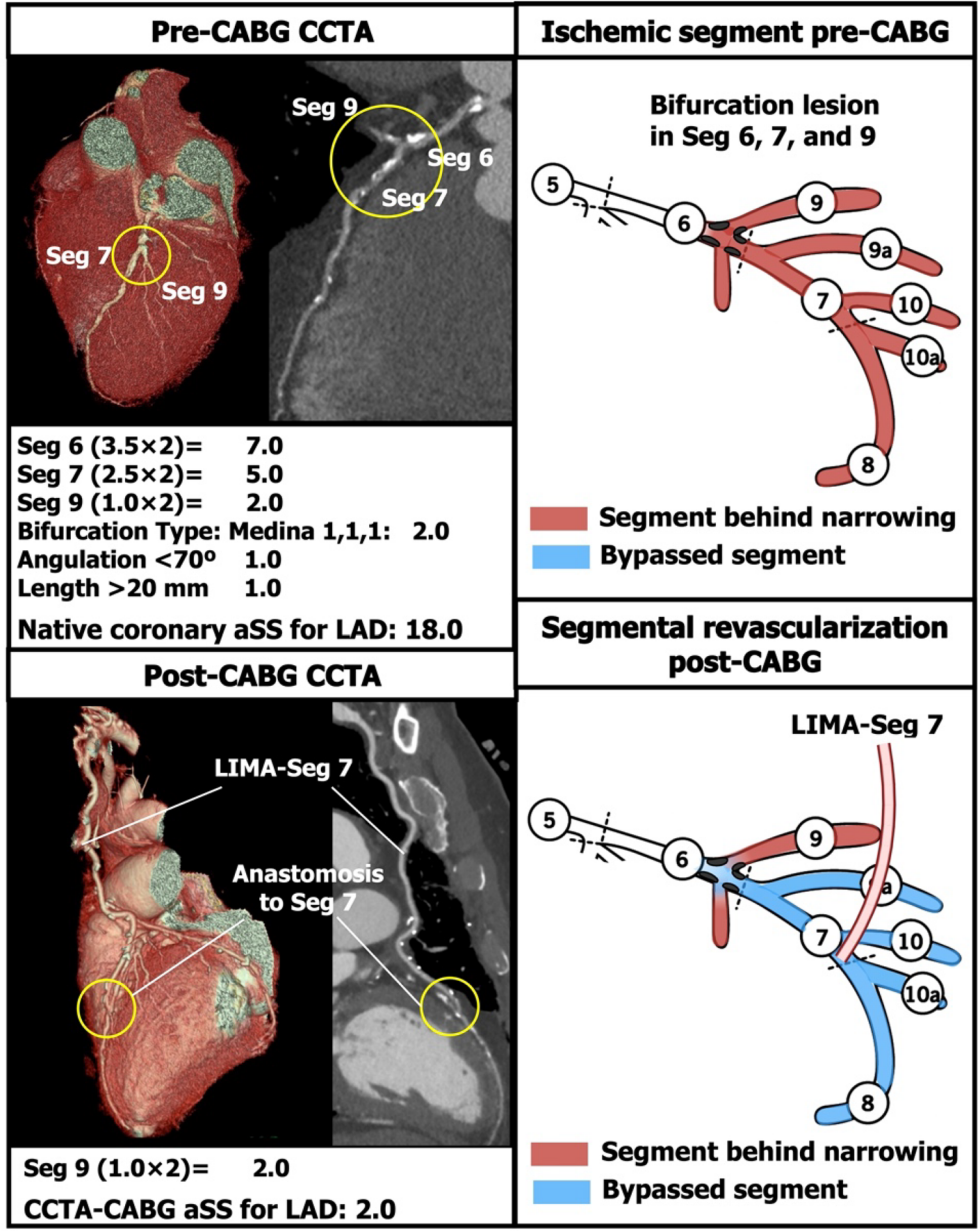
Example of subtracted points from the native coronary aSS in a left anterior descending artery (LAD) with a bifurcation lesion. If the bifurcation lesion had a Medina class of 1-1-1 and was bypassed with a single anastomosis to the distal LAD, then the aSS points for the LAD would be deducted, but not those for the diagonal branch. In this case, the remaining points were (1 × 2) = 2 for the stenosis in the diagonal branch. Abbreviations as in Fig. 1

**Fig. 5:**
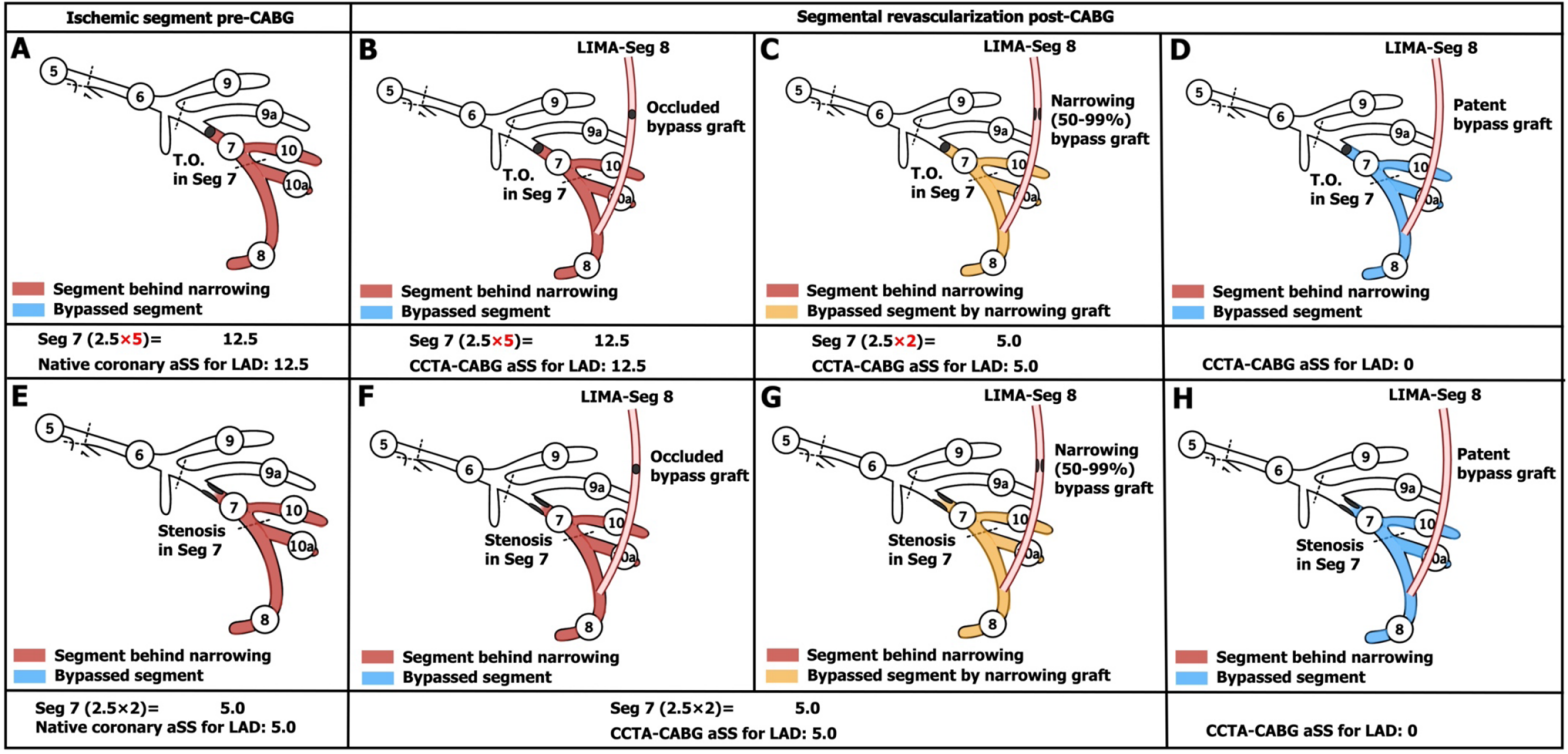
A variety of different scenarios showing the points subtracted from the native coronary aSS when an obstructed or occluded native vessel has an occluded, stenosed or patent bypass graft. In cases of an occluded native vessel with a stenosed bypass graft (Panel C), the segment-weighting factor was reduced from ×5 to ×2 to signify the net improvement in flow to the myocardial bed had improved. Abbreviations as in Fig. 1

2. Whenever the graft and anastomoses were patent, the segment-weighting points of the bypassed coronary segment, including any points for adverse lesion characteristics, were subtracted (Fig. 1).

3. When the vessel (e.g., left artery descending artery [LAD]) proximal to the anastomosis had > 1 stenotic lesion then the aSS points of those coronary segments distal and closest upstream of the anastomosis (segment 7 in Fig. 2) were deducted, whilst the aSS points of the proximal coronary segment (segment 6 in Fig. 2) continued to be included to account for the persistence of the proximal narrowed ischemic segments. Another example is in Fig. 3. If the bifurcation lesion had a Medina class of 1-1-1 and was bypassed with a single anastomosis to one of the daughter vessels (LAD in Fig. 4), then the aSS points for the lesion in the bypassed vessel (segment 6 and 7 in Fig. 4) including points for the characteristic of bifurcation disease was deducted.

4. In the case of an occluded bypass graft, the points of the native aSS were not subtracted (Fig. 5B, F).
5. In cases where the bypass graft had a 50–99% stenosis and was anastomosed on: [1]
  - Obstructive native coronary lesion (50–99%): no segment-weighting points were subtracted since the lesions were considered flow limiting (Fig. 5G);
  - Occluded native coronary lesion (100%): the segment-weighting factor was reduced from × 5 to × 2 since the flow to the myocardial bed had improved (Fig. 5C).

### Reproducibility analysis

To evaluate inter-observer reproducibility, two blinded readers in the independent core laboratory (CORRIB Core Lab, Galway, Ireland) with experience in the interpretation of more than 200 mentored cases of CCTA in clinical research calculated the CCTA-CABG aSS using CCTAs performed 30 days (± 7 days) after CABG in 45 consecutive cases from the FASTTRACK CABG trial (NCT04142021) [9]. Vessel analysis software (CardIQ Xpress 2.0, GE Healthcare) was used for the analysis. The CCTA acquisition is in Supplementary Methods 1 [9]. Ethical approvals from the ethics committee of the Centro Cardiologico Monzino (R1158/20-CCM 1220), University Hospital of Brussels (B1432020000236), University Hospital of Jena (2020-1889-1-BO), and Mount Sinai School of Medicine (FWA#00005656) have been obtained. Each patient has to provide written informed consent as approved by the ethical committee of the respective clinical site.

### Statistical analysis

Continuous variables are presented as mean ± standard deviation (SD) or as median and interquartile range (IQR) depending on their distribution and compared using the Student’s *t*-test. Inter-observer variability (tercile partitioning) was determined with weighted kappa statistics (< 0 none, 0–0.20 slight, 0.21–0.40 fair, 0.41–0.60 moderate, 0.61–0.80 substantial, 0.81–1.00 almost perfect) using linear weights on the native coronary aSS and the CABG-aSS [1]. All statistical analyses were performed using R version 4.1.3 (R Foundation for Statistical Computing, Vienna, Austria) or SPSS version 27.0 (IBM Inc, Armonk, NY, USA).

## Results

### Feasibility of the CCTA-based CABG-aSS

The CCTA-CABG aSS was successfully evaluated in all 45 patients who had a total of 117 bypass grafts, made up of 65 arterial (55.6%) and 52 saphenous vein (45.4%) grafts, and 152 anastomoses.

### Distribution of the CCTA-based CABG aSS

**Fig. 6:**
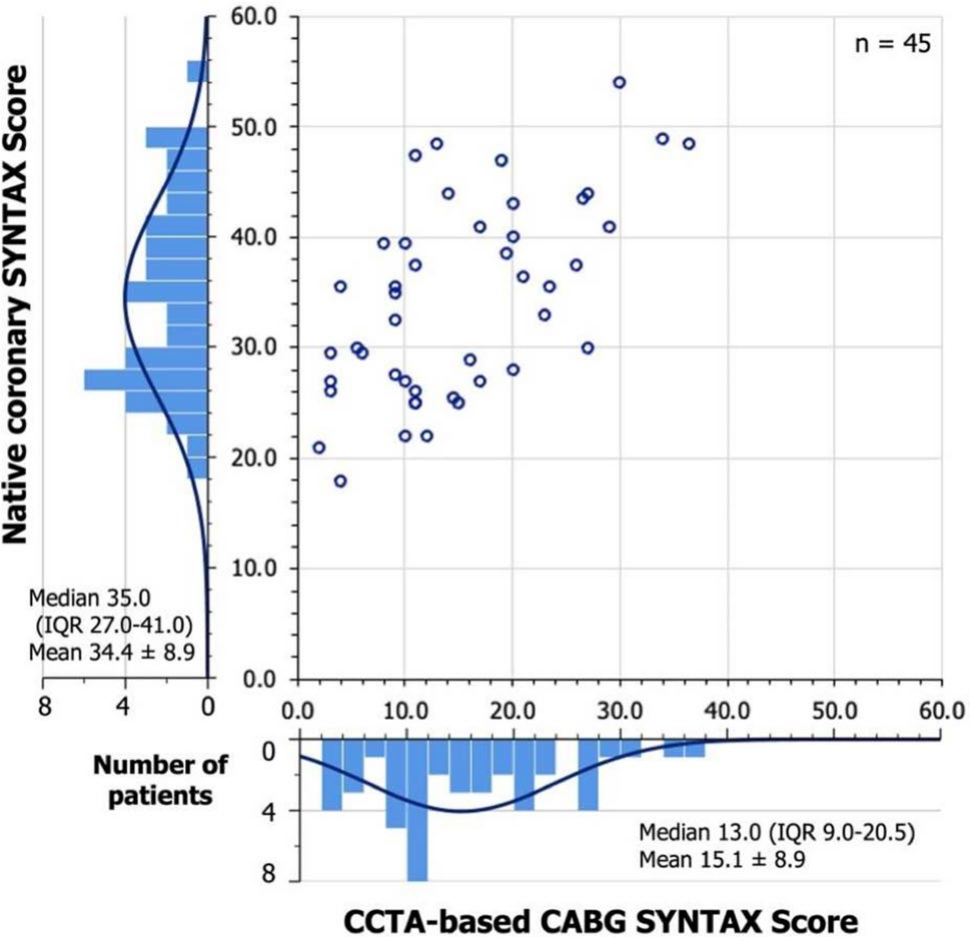
Distribution of the native coronary aSS and CCTA-based CABG aSS. *aSS* anatomical SYNTAX Score, *CABG* coronary artery bypass graft, *CCTA* coronary computed tomographic angiography

 The median CABG aSS was 13.0 (IQR 9.0–20.5, mean 15.1 ± 8.9), with a score > 22 in 22.2% of cases (Fig. 6). The median native coronary aSS on follow-up CCTA was 35.0 (IQR 27.0–41.0, mean 34.4 ± 8.9), with scores > 22 and > 32 in 91.1%, and 55.6% of cases, respectively.

### Reproducibility analysis

The inter-observer level of agreement for the native coronary aSS by tercile partitioning (aSS ≤ 28, 28 < aSS ≤ 39, aSS > 39) was moderate (Kappa = 0.58, 95% CI 0.400–0.758). When the native coronary aSS was stratified based on the tercile of the original SYNTAX trial (i.e., low aSS ≤ 22, intermediate aSS > 22–≤ 32, high aSS > 32), the agreement was substantial (Kappa = 0.67, 95% CI 0.470–0.869). The intraclass correlation (ICC) for inter-observer variability of the native coronary aSS was 0.91 (95% CI 0.83–0.95).

The inter-observer level of agreement in the CCTA-CABG aSS by tercile partitioning (CCTA-CABG aSS ≤ 10, 10 < CCTA-CABG aSS ≤ 19, CCTA-CABG aSS > 19) was substantial (Kappa = 0.61, 95% CI 0.445–0.784). The ICC for inter-observer variability of the CCTA-CABG aSS was 0.92 (95% CI 0.85–0.96). Similarly, when the CCTA-CABG aSS was divided into < 22 or ≥ 22, the agreement was substantial (Kappa = 0.64, 95% CI 0.355–0.925).

## Discussion

The main findings from this study were that the CCTA-CABG aSS was feasible in all study patients, with substantial inter-observer reproducibility.

### Inter-observer reproducibility

Reproducibility analyses for the native aSS, stratified according to terciles of the original SYNTAX trial, and the CCTA-CABG aSS demonstrated substantial inter-observer agreement (Kappa = 0.67 and 0.61, respectively). In previous publications, Kappa values for the inter-observer agreement of the aSS based on ICA and assessed by a core lab ranged from 0.52 to 0.82 [10–12] (Supplementary Table 1), which is similar to that seen for the CCTA-based native aSS in the present study, as well as the CCTA-based CABG-aSS. In our study, each assessment was performed by a single analyst, and as previously reported [10], we accept that our reproducibility may have improved if we had used a consensus opinion of two analysts.

In the previous literature, Kappa of the agreement to stratify the CCTA-based aSS into ≤ 22 or > 22 between sites and the core lab was reported as 0.25 [13]. It was also reported that after advanced training, Kappa of the interobserver agreement to stratify the ICA-based aSS into the tertile of the original SYNTAX trial by interventional cardiologists increased from 0.33 to 0.76 [11]. The experience of readers and training for less experienced readers should be considered for the clinical application.

### Median CABG-aSS

The CCTA-CABG aSS is intended for evaluating and quantifying the completeness of revascularization after CABG, as it reflects the number of coronary segments not adequately bypassed at the time of surgery. In the current population, the median CCTA-CABG aSS was 13.0 (IQR 9.0–20.5, mean 15.1 ± 8.9), with no patient having a score of zero. In the SYNTAX-LE MANS sub-study, those patients with an ICA-based CABG-aSS ≥ 22 post-CABG had significantly higher rates of 5-year death (14.5% vs. 9.1%, log-rank p = 0.012) and the composite of all-cause death, cerebrovascular accident and myocardial infarction (log-rank p = 0.025) compared to those with the score < 22 [14]. In the SYNTAXES (SYNTAX Extended Survival) trial, a higher ICA-based CABG-aSS was associated with a numerically higher risk of 10-year all-cause death (27.8% vs. 14.0%; hazard ratio 2.24, 95% CI 0.95–5.30; log-rank p = 0.058) [15]. Whilst it is anticipated that the CCTA-CABG aSS will have a similar prognostic role, this ultimately needs to be formally investigated in future studies.

### Clinical application of the CCTA-CABG aSS

In the recent AHA/ACC/ACCP/ASPC/NLA/PCNA guidelines for the management of patients with chronic coronary disease, CCTA to evaluate bypass graft in patients with symptoms was recommended as 2a [16]. In addition, in the GRAFFITI (Graft patency after FFR-guided versus angiography-guided CABG) trial, a 1-year follow-up was performed successfully with CCTA in more than 90% of patients, with only a minority requiring ICA (9.8%) [17]. Considering these facts, the use of CCTA for follow-up after CABG and for diagnosis of patients with chest pain who previously underwent CABG in clinical practice will increase.

CCTA-CABG aSS could aid planning pre-procedure and assessment post-procedure as the universal index for the completeness of the revascularization after CABG. Furthermore, when we consider the revascularization for patients with chest pain who previously underwent CABG, this scoring to assess the severity of coronary artery disease could be a useful tool to share the assessment as objective metrics for the Heart team discussion for decision-making.

## Assessment of completeness of revascularization

**Fig. 7:**
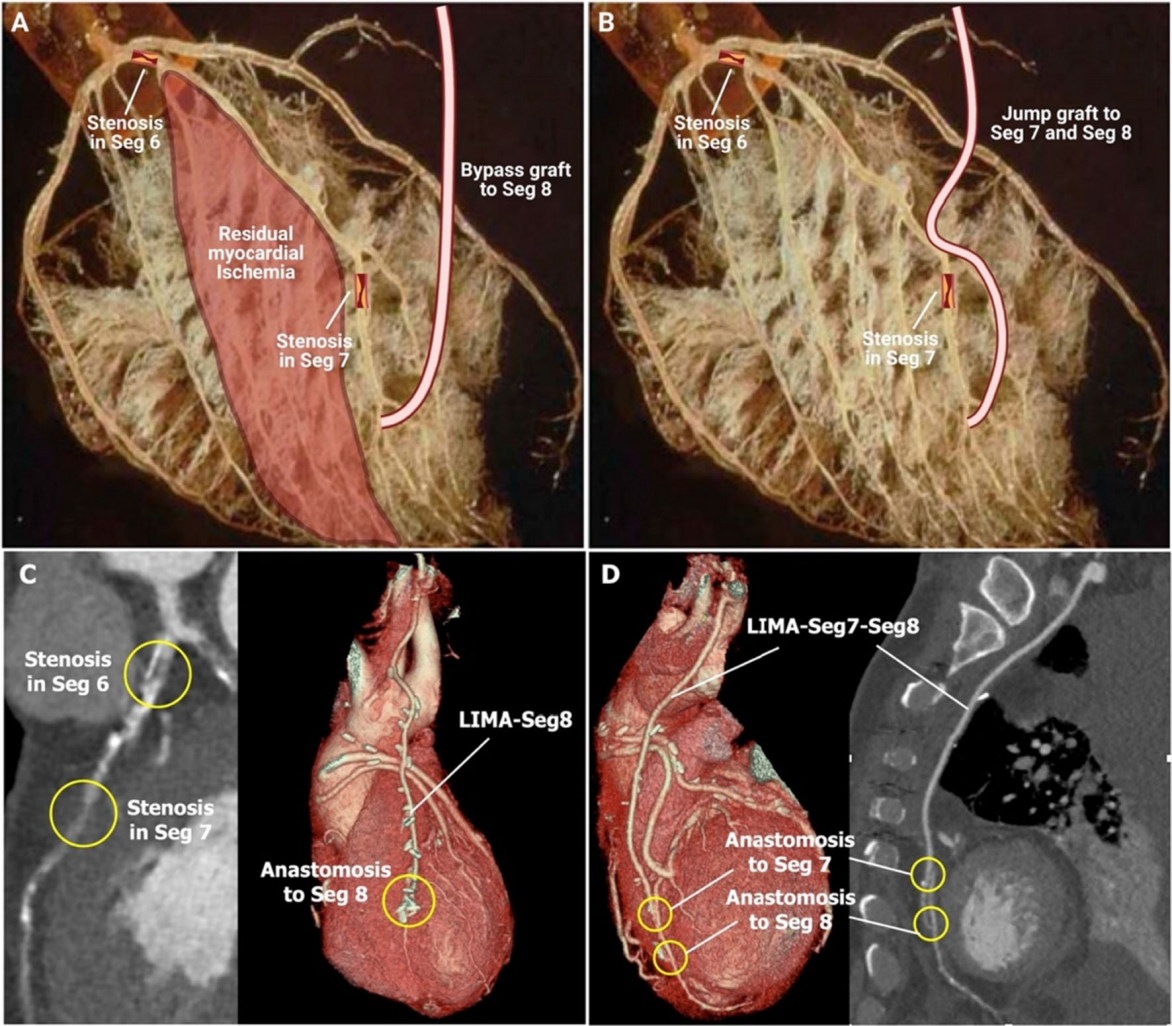
Difference in residual ischemic burden between a single graft and a jump graft on a left artery descending artery (LAD) with serial lesions. Epicardial arteries (> 400 μm) represent only 5% of the volume of the coronary tree. Pre-arterioles, arterioles, and capillaries represent 95% of the remaining coronary volume, and each coronary segment subtends a well-delineated mass-volume of the myocardium. The post-mortem cast of the coronary circulation depicts the abundance of the microvasculature and helps in understanding the difference in residual myocardial ischemia between a single (**A**, **C**) and jump graft (**B**, **D**) for an LAD with serial lesions. *LIMA* left internal mammary artery, *Seg* segment. The picture on Panel A and B was originally published in Journal of Nuclear Medicine (Camici PG, Rimoldi OE. The Clinical Value of Myocardial Blood Flow Measurement. Journal of Nuclear Medicine 2009;50:1076. © 2009 by the Society of Nuclear Medicine, Inc) [19]

 In terms of evaluating the completeness of revascularization, previous ICA-based definitions were ambiguous in cases of sequential lesions, diffuse disease, and bifurcations. Given the high complexity of CAD in the FASTTRACK CABG study (mean baseline CCTA-derived aSS: 35.6 ± 11.5, in the first 57 patients) [18], the residual aSS should fully reflect the completeness of revascularization, accounting for the number of obstructed coronary segments not anastomosed by functioning bypass grafts because of anatomical reasons [e.g., a distal left circumflex artery (LCX) located in the atrioventricular groove and not accessible to the surgeon]; the unavailability of graft material (e.g., previous varicose vein surgery), or obstruction/occlusion of the graft. For example, in the case of sequential lesions in segments 6 and 7, the graft could be anastomosed on either only segment 8 (Fig. 7A, C), or on segment 7 and 8 as a jump graft (Fig. 7B, D). The previous definitions [14] do not differentiate these two approaches, while it is evident in the present case that a jump graft is superior to an end-to-side single graft, in supplying blood and reducing the ischemic burden of the anterior wall. Therefore, in the near future, we intend to utilize the CCTA-CABG aSS as an objective measure of the level of surgical revascularization and residual CAD.

### Prospects of the quantitative assessment of myocardial perfusion after CABG

A comprehensive and universally accepted definition of complete revascularization after CABG and PCI is a major and somewhat utopic challenge since it depends very much on the modality used for the assessment: vascular and segmental, involving patency and narrowing; or myocardial and segmental, investigating viable myocardium at risk of ischemia. The ultimate scientific goal is to combine these two facets of mechanical revascularization. In our current methodological approach, the scientific target is more specifically vascular and segmental, without speculating on the amount of flow needed by the myocardium subtended by a native vessel or graft. The data from 1,162 patients who underwent off-pump CABG for three-vessel disease showed that functional complete revascularization, defined as constructing bypass grafts to coronary artery territories where ischemia was identified by pre-operative myocardial SPECT (single-photon emission computed tomography), had a significant impact on long-term survival [20], whereas recent clinical trials demonstrated similar short-term outcomes between patients with fractional flow reserve (FFR)-guided and ICA-guided CABG [17, 21].

In the present assessment, theoretical weighting factors established by Leaman et al. and based on literature existing at the time of the score’s design were used, and these constitute the foundation of the aSS [22]. In this seminal description, the average coronary blood flow was assumed to be 96 ml per 100 g of left ventricular muscle. Based on the difference in myocardial mass, it was assumed that the LAD carries approximately 3.5 times and the LCX 1.5 times as much blood as the RCA (right coronary artery). The LAD, LCX, and RCA were further subdivided, and weighting factors were theoretically assigned to each coronary sub-segment. At that time, the assumptions of myocardial mass were based on studies which assessed roentgenograms of ventricular myocardium post-mortem [23], or clearance rates of radioactive gas from the myocardium of animals and man [24, 25].

**Fig. 8:**
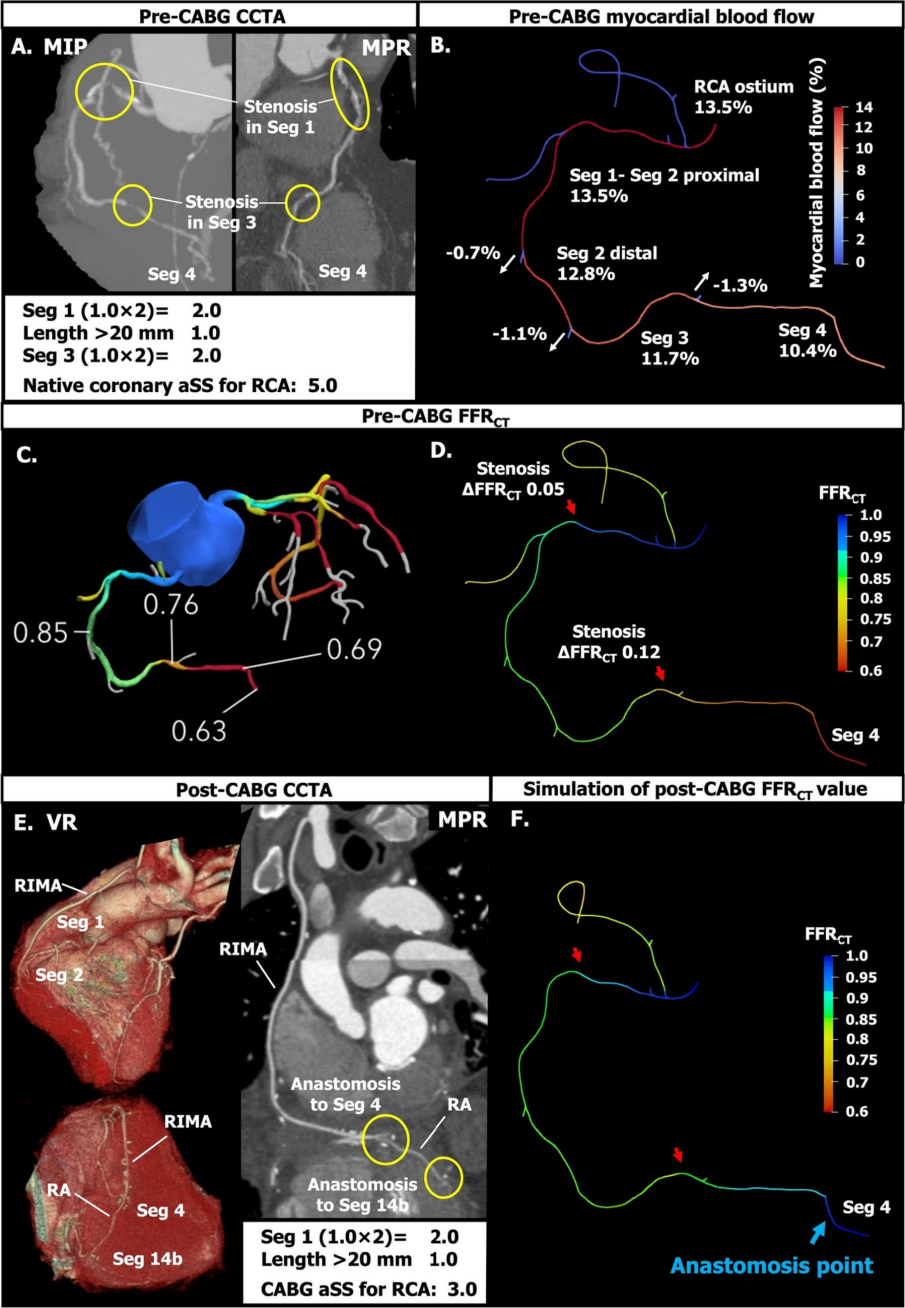
Assessment of myocardial blood flow and FFR_CT_ after CABG. **A** Pre-CABG maximum intensity projection (MIP) and curved multiplanar reconstruction (MPR) images: yellow circles indicate significant stenoses in segments (Seg) 1 and 3. **B** Simulation of myocardial blood flow to the left ventricle yields a quantitative assessment of the myocardium at risk, and the area subtended by each coronary segment and its side branches. **C** and **D** FFR_CT_ values before CABG. **E** Post-CABG volume rendering (VR) and MPR. **F** Post-CABG FFR_CT_ based on a simulation of both flows from the aorta and bypass graft anastomosed on Seg 4

 Although the prognostic value of the aSS in PCI has been repeatedly demonstrated over more than one decade, data on residual aSS post-CABG and its prognostic value are scarce [14, 15]. The present proposal is a pragmatic attempt to modify the ICA-aSS post-CABG to use CCTA, which may serve as a reasonable tool during the current transition period where technological advances in CCTA hardware are occurring and where new software assessing volume flow are emerging and being validated versus PET (positron emission tomography). Indeed, the need for measuring regional flow and the myocardium at risk becomes increasingly important for decision-making between pharmacological treatment and mechanical revascularization, be it percutaneous or surgical. Ultimately, the historical and theoretical weighting scores universally applied to every patient need to be replaced by parametric measurements specific to each vessel sub-segment and subtended myocardium at risk, and that type of analysis must be individualized for each patient (i.e., patient-specific weighting factors). Quantitative simulated perfusion of the myocardium might be obtained from CCTA and related to FFR_CT_ (FFR derived from CCTA), as illustrated in Fig. 8. Patient-specific myocardium at risk of each target lesion might help achieve “reasonable revascularization” with limited graft resources [26–28].

The prognostic value of the aSS stemmed from prospective testing in the landmark Syntax trial [10], and was confirmed more than a decade later by a pooled analysis of four randomized trials involving left main with one, two, or three vessel disease [29].

If the method of quantifying the myocardium at risk and FFR_CT_ becomes common practice, the challenge will be to prospectively re-evaluate and re-establish a new scale of risk assessment related to the decision-making process.

### Limitations

Anatomical evaluation of residual disease after CABG is still currently based on historical and theoretical weighing scales related to the presence of a narrowing in a specific coronary segment, regardless of the caliber, length, and amount of myocardium actually supplied by that specific vessel [22]. CCTA-based physiological assessment using FFR_CT_ and simulated blood flow measurements might allow better evaluation of flow-limiting lesions before and after CABG [30].

The assessment of the area of ischemic myocardium using a purely anatomical evaluation remains challenging. After graft placement, antegrade coronary flow through and beyond the native coronary artery stenosis can be reversed [31, 32]. Competitive flow from the native coronary artery into the bypass graft remains an investigational issue [33]. Results of RCTs have demonstrated similar graft failure rates and short-term clinical outcomes between FFR-guided and angiography-guided CABG [17, 21]. Recently, the concept of imbalance between the global luminal volume of the supply vessel and the mass of myocardium supplied by that vessel, the so-called V/M ratio, has been implicated as one of the causes of persisting ischemic symptoms when a graft is anastomosed in the middle of a long and diffusely diseased vessel [34–37].

## Conclusions

The CCTA-CABG aSS was feasible in all patients who underwent CABG for complex coronary artery disease with substantial inter-observer reproducibility, and therefore can be used to quantify the completeness of revascularization after CABG. The potential of a threshold residual value that identifies high-risk patients post-CABG should be investigated in prospective studies.

### Supplementary Information

Below is the link to the electronic supplementary material.

Supplementary material 1 (DOCX 36 kb)
