# Supplementary material for: CCTA-based CABG SYNTAX Score: a tool to evaluate completeness of coronary segment revascularization after bypass surgery
Source: Int J Cardiovasc Imaging. 2023 Nov 3;39(12):2531–43. doi: 10.1007/s10554-023-02978-9 (PMC10692266; doi:10.1007/s10554-023-02978-9)
Supplement: Supplementary file 1 — Supplementary material 1 (DOCX 36 kb) [file 10554_2023_2978_MOESM1_ESM.docx]

**CCTA-based CABG SYNTAX Score: A Tool to Evaluate Completeness of Coronary Segment Revascularization after Bypass Surgery**

**Supplementary material**

**Supplementary Methods 1. CCTA acquisition**

At 30-day follow-up, CCTA was performed to assess the native coronary arteries and bypass grafts. Therefore, in addition to the triggered acquisition of CCTA for coronary artery, a second non-gating acquisition was performed.

CCTA was obtained using the 256-slice GE Healthcare Revolution CT scanner. Standard acquisition techniques were used. Heart rate modulation was performed for heart rates >60/min using metoprolol (oral or intravenous). Nitrates were administrated prior to image acquisition, with beta-blockers to avoid reflex tachycardia/vasoconstriction. Tube settings were adjusted depending on patient body mass index (100 or 120 kV). Prospective ECG-triggered one-beat scan mode was used to reduce radiation doses. Images were reconstructed using 0.625 mm slice thickness and standard kernel reconstruction. Detailed acquisition protocol is described elsewhere (1).

**Supplementary Table 1.** **Reproducibility of ICA-based SYNTAX Score, ICA-based CABG SYNTAX Score, and CCTA-based SYNTAX Score**

| **Trial/Author** | **Year** | **Comparison** | **Results** |
| --- | --- | --- | --- |
| **ICA-based SYNTAX Score** | | | |
| SYNTAX/  Serruys et al. (2) | 2009 | Intra-observer variability for ICA-derived SYNTAX Score tertile: ≤22, >22-≤32, >32 | Kappa 0.61 |
|  |  | Inter-observer variability for ICA-derived SYNTAX Score tertile: ≤22, >22-≤32, >32 | Kappa 0.52 |
| SYNTAX/  Garg et al. (3) | 2010 | Intra-observer variability for ICA-derived SYNTAX Score tertile: ≤22, >22-≤32, >32 | Kappa 0.51 |
| Généreux et al. (4) | 2011 | Inter-observer variability for ICA-derived SYNTAX Score by core lab tertile: ≤22, >22-≤32, >32 | After basic training: Kappa 0.82 (95% CI 0.72-1.00)  After advanced training: 0.84 (0.76-1.00) |
|  |  | Inter-observer variability for ICA-derived SYNTAX Score by interventional cardiologist group tertile: ≤22, >22-≤32, >32 | After basic training: Kappa 0.33 (95% CI 0.18-0.44)  After advanced training: 0.76 (0.64-1.00) |
| ACUITY (5) | 2012 | Inter-observer variability for ICA-derived SYNTAX Score | Kappa 0.76 (95% CI 0.64-1.00) |
| **ICA-based CABG SYNTAX Score** | | | |
| SYNTAX-LE MANS (6) | 2013 | Intra-observer variability for ICA-derived (15-month) native SYNTAX Score tertile | Kappa 0.70 (95% CI 0.50-0.91) |
|  |  | Intra-observer variability for ICA-derived (15-month) CABG SYNTAX Score tertile | Kappa 0.70 (95% CI 0.50-0.90) |
| **CCTA-based SYNTAX Score** | | | |
| Papadopoulou et al. (7) | 2013 | Intra-observer variability for CT-SYNTAX Score tertile: ≤9, >9-≤22, >22 | Kappa 0.80 (95% CI 0.67-0.94) |
| SYNTAX III  /Collet et al. (8) | 2018 | CT vs. ICA-derived SYNTAX Score tertile: ≤22, >22-≤32, >32 | Kappa 0.33 (95% CI 0.23-0.42) |
| SYNTAX III  /Katagiri et al. (9) | 2021 | CT-derived anatomiocal SYNTAX Score by sites vs. core lab: ≤22 or >22 | Kappa 0.25 (95% CI 0.09-0.41) |
|  |  | Functional SYNTAX Score based on CCTA and FFR_CT_ by sites vs. core lab: ≤22 or >22 | Kappa 0.35 (95% CI 0.20-0.50) |

CABG = coronary artery bypass graft; CCTA = coronary computed tomographic angiography; CI = confidence intervals; FFR_CT_ = fractional flow reserve derived from coronary computed tomographic angiography; ICA = invasive coronary angiography.

**References**

1. Kawashima H, Onuma Y, Andreini D et al. Successful coronary artery bypass grafting based solely on non-invasive coronary computed tomography angiography. Cardiovascular Revascularization Medicine 2021.

2. Serruys PW, Onuma Y, Garg S et al. Assessment of the SYNTAX score in the Syntax study. EuroIntervention 2009;5:50-6.

3. Garg S, Girasis C, Sarno G et al. The SYNTAX score revisited: a reassessment of the SYNTAX score reproducibility. Catheter Cardiovasc Interv 2010;75:946-52.

4. Généreux P, Palmerini T, Caixeta A et al. SYNTAX score reproducibility and variability between interventional cardiologists, core laboratory technicians, and quantitative coronary measurements. Circ Cardiovasc Interv 2011;4:553-61.

5. Généreux P, Palmerini T, Caixeta A et al. Quantification and impact of untreated coronary artery disease after percutaneous coronary intervention: the residual SYNTAX (Synergy Between PCI with Taxus and Cardiac Surgery) score. J Am Coll Cardiol 2012;59:2165-74.

6. Farooq V, Girasis C, Magro M et al. The CABG SYNTAX Score - an angiographic tool to grade the complexity of coronary disease following coronary artery bypass graft surgery: from the SYNTAX Left Main Angiographic (SYNTAX-LE MANS) substudy. EuroIntervention 2013;8:1277-85.

7. Papadopoulou SL, Girasis C, Dharampal A et al. CT-SYNTAX score: a feasibility and reproducibility Study. JACC Cardiovasc Imaging 2013;6:413-5.

8. Collet C, Onuma Y, Andreini D et al. Coronary computed tomography angiography for heart team decision-making in multivessel coronary artery disease. European Heart Journal 2018;39:3689-3698.

9. Katagiri Y, Andreini D, Miyazaki Y et al. Site vs. core laboratory variability in computed tomographic angiography-derived SYNTAX scores in the SYNTAX III trial. Eur Heart J Cardiovasc Imaging 2021;22:1063-1071.
